# Aortic valve disease: Novel animal models for advancing our understanding of the underlying mechanisms

**DOI:** 10.1002/ame2.70294

**Published:** 2026-09-27

**Authors:** Liya Hou, Xue Liu, Wangxing Hu, Xianbao Liu, Wei Zhu

**Affiliations:** ^1^ Department of Cardiology The Second Affiliated Hospital, School of Medicine, Zhejiang University Hangzhou China; ^2^ State Key Laboratory of Transvascular Implantation Devices Hangzhou China; ^3^ Heart Regeneration and Repair Key Laboratory of Zhejiang Province Hangzhou China; ^4^ Transvascular Implantation Devices Research Institute Hangzhou China; ^5^ The First Affiliated Hospital of Zhejiang Chinese Medical University (Zhejiang Provincial Hospital of Chinese Medicine) Hangzhou China; ^6^ The First Clinical Medical College of Zhejiang Chinese Medical University Hangzhou China

**Keywords:** animal models, aortic stenosis, aortic valve calcification, aortic valve disease, aortic valve hereditary dysplasia, aortic valve malformation

## Abstract

The global public health burden of aortic valve disease (AVD) is growing due to its high mortality rate and lack of suitable pharmaceutical therapies in clinical practice, a challenge that is largely attributable to our poor understanding of its complicated underlying mechanisms. A better understanding of the pathological processes underlying AVD is urgently needed to identify novel therapeutic options. Appropriate animal models are emerging as vital tools for basic and translational research, and the development of new models and the important information they have provided has significantly aided our research. However, different animal models have unique limitations in their ability to mimic human AVD pathology. In this review, we summarize the available AVD animal models and provide a detailed description of the major principles underlying each animal model, including methodology, pathological features, and potential use and application in AVD research. Both the advantages and disadvantages of specific models are also carefully highlighted; thus this comprehensive review provides a valuable animal model reference for both basic and preclinical researchers.

## Graphical abstract

Due to the complex pathophysiology of aortic valve disease (AVD) and the resulting lack of effective pharmacotherapy, existing animal models are valuable research tools, but each has its own strengths and limitations. This review systematically surveys the major AVD models, compares their induction methods, pathological features, advantages and disadvantages, applicable scenarios, and their use in basic and preclinical research, and aims to provide researchers with a comprehensive reference for model selection to support mechanistic studies and therapeutic target discovery.

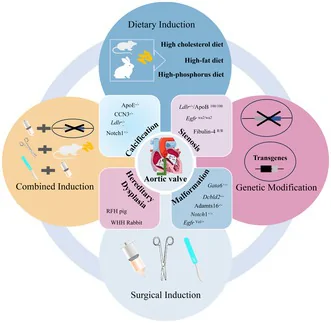

## 1 INTRODUCTION

AVD is a valvular condition characterized by calcification, stenosis, malformation and hereditary dysplasia, and it accounts for more than 61% of all valvular heart disease (VHD).^1^ Over the past decade, the global incidence of AVD has increased by 155%, and thus management of the disease has emerged as a critical public health challenge.^2^ Although transcatheter aortic valve implantation (TAVI) has revolutionized severe aortic stenosis management, surgical aortic valve replacement remains the gold standard for younger patients and those requiring concurrent cardiac surgery.^3^ However, persistent complications such as bioprosthesis degeneration and paravalvular leakage (PVL) affect up to 20% of TAVI patients^4^ and pharmacotherapies for halting early calcification are lacking, making the development of targeted molecular interventions and tissue engineering solutions an urgent matter.

Physiologically, the aortic valve features a collagenous trilaminar structure comprising the fibrosa, spongiosa and ventricularis. Within the extracellular matrix (ECM), interactions between valvular interstitial cells (VICs) and valvular endothelial cells (VECs) ensure lifelong hemodynamic stability for the valve.^5^, ^6^ Disruption of these interactions leads to AVD, which typically manifests as stenosis, calcification, or regurgitation. The onset of AVD is generally characterized by endothelial dysfunction, which can be triggered by pathological changes to hemodynamic processes such as the oscillatory shear stress observed in bicuspid valves.^7^ This dysfunction is often compounded by systemic conditions, including hypertension and chronic kidney disease, which produce proinflammatory signals that collectively drive the osteogenic transformation of VICs^8^ through three intersecting axes: the inflammation‐fibrosis axis,^9^, ^10^ the calcification‐metabolism axis^11^, ^12^ and the ECM‐mechanotransduction axis.^13^ Despite progress in understanding the pathogenesis of AVD, the substantial mechanistic heterogeneity among forms of the disease with different etiologies complicates the development of a universal pathophysiological framework. Resolving this translational bottleneck requires integrating methods such as single‐cell spatial transcriptomics and biomechanical modeling to generate the integrated data needed to identify context‐specific signaling networks and thereby better elucidate the mechanisms of AVD.

Animal models are a key tool for understanding the complex interactions between hemodynamic forces and biochemical signaling pathways, such as those involving TGF‐β and BMP.^9^ Current research employs different types of animals, such as pigs,^14^ mice, and sheep.^15^ However, these models exhibit unique pathological limitations that restrict their translational potential, requiring rigorous data interpretation. Specifically, calcification in rodents occurs much faster than in humans, and large animal models often lack high‐resolution molecular profiles. Methodologically, current surgical,^16^ dietary^17^ and genetic induction^18^ methods are unable to fully recapitulate the human disease spectrum from early dysfunction to fatal calcification. The goal of this article is to review existing animal models of AVD described in the published literature with a focus on how they are applied to provide researchers with comprehensive information or guidance for better understanding the underlying disease mechanisms and developing novel therapeutic targets. Specifically, we summarize their modeling principles, methodology, pathological features, unique features, and advantages and potential disadvantages (Figure 1). The substantial utility of animal models in AVD research is also discussed, with a particular emphasis on opportunities for further improvement.

**FIGURE 1:**
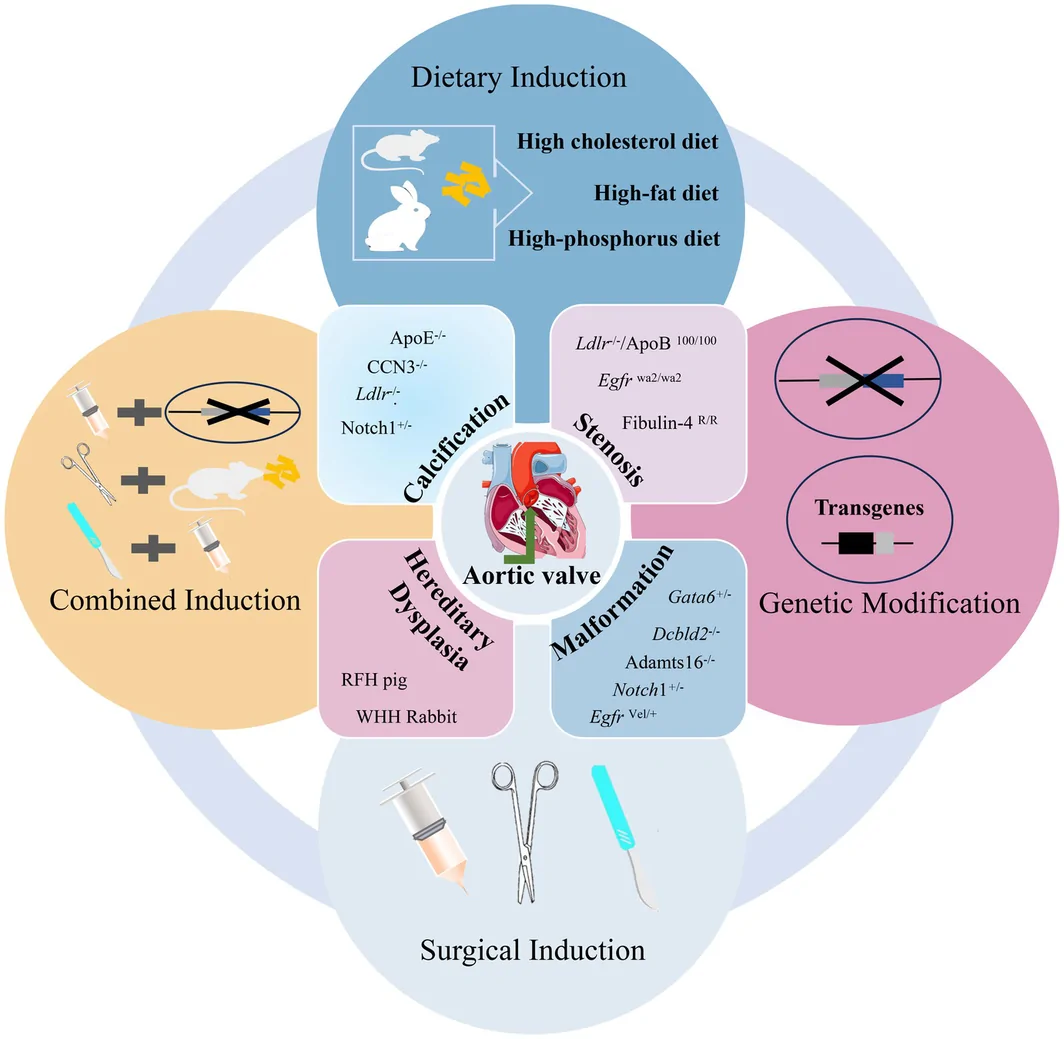
Schematic overview of aortic valvular heart disease: content and methodology.

## 2 ANIMAL MODELS

The pathogenesis of AVD involves interactions among factors such as aging, hypertension, obesity,^19^ and diabetes.^20^ These risk factors drive calcific degeneration and congenital anomalies and make AVD the most common VHD in clinical practice.^21^ This section provides a critical review of the foundation and preclinical animal models of AVD to elucidate the underlying mechanism of the disease and obtain therapeutic insights into it.

### 2.1 Animal models of aortic valve calcification

CAVD progresses from early subclinical leaflet thickening to severe calcific stenosis and is characterized by chronic inflammation, fibrosis, and ectopic mineralization in which calcium deposits stiffen the valves and obstruct left ventricular outflow.^22^, ^23^ Pathologically, the pathogenesis of CAVD begins with endothelial dysfunction involving lipid deposition and inflammatory infiltration^24^, ^25^ followed by fibrotic remodeling, VIC activation, osteogenic differentiation and calcification.^24^, ^26^ Specifically, oxidized low‐density lipoprotein (oxLDL) and lipoprotein(a) accumulate within the valve leaflets,^27^ causing immune cell recruitment and triggering the release of TGF‐β and TNF‐α to drive VIC osteogenesis.^28^ Concurrently, endothelial injury promotes neoangiogenesis, allowing further influx of circulating lipids and inflammatory cells and accelerating aortic valve calcification.^27^, ^28^, ^29^ Mechanistically, oxLDLs disrupt Ca^2+^ homeostasis and induce endoplasmic reticulum (ER) stress via the PERK/ATF4 and IRE1α/XBP1s pathways, increasing the levels of osteocalcin and Runx2.^30^ Furthermore, endothelial damage exposes collagen and VCAM‐1, activating platelets to release ADP. This purinergic signaling induces autotaxin (ATX) expression in VICs, generating lysophosphatidic acid to accelerate valvular calcification.^31^

Various animal models currently in use provide the common basis for studying CAVD, as they can link experimental verification or information with clinically driven questions and needs. This review provides detailed information regarding the different species‐based animal models used to understand the pathophysiology of human CAVD. We have identified numerous relevant studies and summarize the various approaches to CAVD animal research therein in the following text.

#### 2.1.1 Dietary induced CAVD models

A high‐cholesterol diet (HCD) induces hypercholesterolemia, which is characterized by cholesterol deposition and microcalcification foci that lead to vascular stiffening and hemodynamic disruption. Rajamannan et al.^32^ first demonstrated this phenomenon in rabbits, linking HCD to endothelial injury and osteogenic transformation. Researchers have subsequently reported that a high‐fat/high‐carbohydrate diet (HFD) can also induce AVD, triggering hyperlipidemia and systemic chronic inflammation via abnormal lipid metabolism.^33^ Moreover, dysregulated adipokines promote atherosclerosis‐like valvular fibrosis.^24^, ^34^ Mechanistically, an HCD initiates valvular disease primarily through oxLDL‐mediated endothelial toxicity, whereas the mechanisms of action of an HFD involve systemic low‐grade inflammation. Although both models follow an inflammation‐to‐calcification pathway, they feature distinct metabolic profiles, allowing the simulation of specific CAVD stages.

Unlike wild‐type C57BL/6 mice, which develop only mild stenosis on an HCD, apolipoprotein E‐deficient (ApoE^−/−^) mice exhibit spontaneous hypercholesterolemia that worsens when they are fed an HCD or an HFD.^33^ Table 1 summarizes the induction methods and phenotypes of commonly used hypercholesterolemic mouse CAVD models from relatively recent studies. In ApoE^−/−^ mice, disease progression is characterized by the upregulation of osteogenic markers, calcification and valve thickening.^30^, ^35^ In the early stages, the pathogenesis of the disease involves oxLDL‐mediated cytosolic Ca^*2+*^ accumulation, which triggers ER stress, myofibroblast activation, and macrophage infiltration.^30^ NF‐κB signaling subsequently drives VIC osteogenesis, reducing the aortic valve area (AVA).^30^ Notably, models by Guo et al.^35^ and Yu et al.^36^ demonstrated severe fibrosis and increased transvalvular gradients without systemic metabolic or cardiac dysfunction.^35^, ^36^ These phenotypic variants largely stem from differences in animal age, with older animals better reflecting advanced disease stages. Similarly, HCD‐fed Sprague–Dawley rats can recapitulate the murine pathology but display more pronounced elevations in LDL‐C and IL‐6 levels.^37^ These rat models, constructed via subcutaneous bioprosthetic implantation, are limited primarily to studying immune responses and recellularization. Because the sites of implantation lack essential hemodynamic forces, the translational relevance of the results of these models is significantly hindered by potential confounding effects on tissue regeneration.^17^, ^38^ Conversely, in rabbit models, human CAVD can be effectively reflected through a combination of an HCD and vitamin D2 supplementation.^26^ After 12–16 weeks, application of this dual‐induction regimen results in severe hyperlipidemia and atherosclerotic lesions.^30^, ^39^ Pathologically, macrophage infiltration precedes collagen accumulation and ECM disorganization, among other typical pathological features (Table 1).^39^ Compared with induction with either diet alone, dual induction more effectively mimics human CAVD pathophysiology.

**TABLE 1:** High‐cholesterol diet induced animal models of CAVD.

Species	Strain/gene	Age/gender/weight	Method		Phenotypes						References
			Dietary components	Feeding time	Pathological phenotypes	Molecular markers	Ultrasonic cardiogram	Metabolic parameters	Hemodynamic parameters	Others	
Mice	ApoE^−/−^	8 weeks old	A 0.25% cholesterol diet	24 weeks	Calcification α‐SMA upregulation. ER stress markers (KDEL, CHOP) elevated Osteogenic markers (osterix, Runx2, osteocalcin) increased Macrophage infiltration (RAM11, F4/80 elevated)	/	AVA and AVA index reduced	Serum cholesterol and LDL levels elevated	/	/	[30]
	ApoE^−/−^ (C57BL/6J background)	6 weeks old /male	A 0.2% cholesterol diet	24 weeks	Valvular thickening Fibrosis Calcification	Osteogenic marker(Runx2, Osterix) upregulated	Peak aortic jet velocity and pressure gradient elevated	No change in serum glucose and lipids	/	/	[35]
		6–8 weeks old	0.2% cholesterol diet	24 weeks	Calcification Leaflet thickening	/	Peak transvalvular jet velocity increased AVA and AVA index decreased	No change in glucose or lipid levels	No change in cardiac structure, function, and hemodynamics	/	[36]
Rats	Sprague–Dawley rats	2 months old	A 1.25% cholesterol and 0.5% cholic acid diet	12 weeks	Cellular infiltration	/	/	LDL‐C levels elevated at week 4 IL‐6 levels elevated at week 5, peakedat week 12	/	Calcium content elevated	[37]
Rabbits	New Zealand white rabbits	Male/weighing 2.5–3.0 kg	A 0.5% cholesterol diet with 50 000 IU of vitamin D2 daily	12 weeks	ER stress markers (p‐PERK, p‐IRE1α, CHOP) elevated Osteogenic markers (osterix, Runx2, osteocalcin) upregulated	/	/	Serum cholesterol and LDL levels elevated	/	/	[30]
		12 weeks old/male /weighing approximately 1.83 kg	0.3% cholesterol diet plus vitamin D_2_ (25 000 U/2.5 kg daily)	16 weeks	Leaflet thickening Calcific foci Cell/lipid infiltration Collagen accumulation Matrix disorganization (collagen, GAGs) Macrophage infiltration(RAM11 elevated) Ventricular‐side αSMA upregulation	/	/	/	/	/	[39]
		4 weeks old/ male	A 0.5% cholesterol diet with 25 000 IU of vitamin D2 daily	12 weeks	Leaflet thickening Tissue fibrosis Calcification Macrophage infiltration	/	AVA decreased	/	Aortic velocity (Vmax) and mean pressure gradients increased	/	[40]

Table 2 summarizes contemporary HFD models. In wild‐type C57BL/6 mice, an HFD induces early‐stage CAVD,^41^ which is further exacerbated by supplementation with 1.2% choline.^42^ ApoE^−/−^ mice bred on a C57BL/6J background are preferred for studying age‐dependent, progressive phenotypes, as their early pathogenesis involves inflammatory cytokine release, protective GLA suppression,^43^ and NF‐κB/NLRP3 activation, all of which upregulate the expression of calcification markers.^44^, ^45^ Recently, in vivo optical imaging has offered an alternative monitoring modality to echocardiography, potentially reducing confounding hemodynamic effects.^46^ The macrophage marker CD68 is detectable in the aortic valve at 12–36 weeks,^46^ while the endothelial‐to‐mesenchymal transition (EndMT) occurs during calcification.^47^ After 8 weeks, use of an HFD disrupts lipid profiles in the later stages of CAVD.^47^ HFD‐derived advanced glycation end products (AGEs) further promote calcification via AGE‐RAGE and TGF‐β1/BMPR2 signaling.^47^ Placement on a chronic HFD (40 weeks) induces Wnt/β‐catenin activation and apoptosis and reduces the ejection fraction.^47^ Moreover, the addition of 1.5% adenine accelerates osteogenesis through hyperuricemia‐induced HIF‐α activation.^48^ Similar hemodynamic deterioration has been shown to occur in HFD‐fed New Zealand white rabbits by week 12.^26^ Ultimately, because HFD feeding alone rarely recapitulates end‐stage human CAVD, complementary genetic strategies are necessary, potentially making these models inefficient for evaluating specific lipid‐lowering therapies.^49^

**TABLE 2:** High‐fat diet induced mouse models of CAVD.

Species	Strain/gene	Age/gender/weight	Method			Phenotypes				References
			Dietary components	Feeding time	Pathological phenotype	Molecular markers	Ultrasonic cardiogram	Metabolic parameters	Hemodynamic parameters	
Mice	C57BL/6 mice	3–5 months old/ male	HFD: 24% fat, 41% carbohydrates and 24% protein	16 weeks	Aortic valve leaflet thickening OxLDL deposition	BMP‐2 levels elevated	/	Plasma oxLDL elevated	/	[41]
	C57BL/6 mice	16–18 months old	HFD: 21% fat, 0.15% cholesterol HFCD: 21% fat, 0.15% cholesterol, 1.2% choline	16 weeks	Aortic valve thickening in HFCD compared to HFD	BMP‐2 levels elevated	/	/	/	[42]
	ApoE^−/−^(C57BL/6J background)	3 weeks old	HFD: 42% fat, 0.25% cholesterol	24 weeks	Aortic valve calcification factors Kla, H3K9la, and p300 elevated Valve thickening	/	/	/	/	[45]
				16 weeks	Calcium deposition increased	/	Peak jet velocity increased	/	/	[50]
		3 weeks old/male			Aortic valve leaflet thickening Calcium deposition	GLA expression reduced	/	/	/	[43]
				16–24 weeks	Aortic valve leaflet thickening Calcium deposition	NLRP3 expression increased	/	/	/	[44]
		4–5 weeks old/male	Western diet:0.15% cholesterol and 40% fat	12 weeks, 24 weeks, and 36 weeks	12 weeks:no valve calcification 24 to 36 weeks: calcification increased and progressive leaflet thickening Macrophage marker CD68 detected at 12 weeks, peaked at 24 weeks, declined by 36 weeks	/	24–36 weeks: leaflet separation decreased 36 weeks: peak transvalvular flow velocity increased	/	/	[46]
		6 weeks old/male	HFD: 42% fat, 0.2% cholesterol	8, 16 and 24 weeks	Calcium granule appeared at leaflet edge Progression to linear marginal deposition Invasion into valve interior Preferential deposition on aortic side	CML and RAGE elevated. CML progression from surface to subsurface VE‐cadherin downregulation (nadir at 24 weeks); α‐SMA upregulation at 16–24 weeks TGF‐β1 high at 8 weeks, decreased by 16 weeks, lowest at 24 weeks BMPR2 downregulation ALP up at 8 weeks, peaked at 16 weeks, down by 24 weeks α‐SMA/ALP double‐positive areas at leaflet edge (8 weeks)	Transvalvular velocity elevated	Serum levels of LDL, TC, and TG elevated Serum HDL reduced	/	[47]
		7–8 weeks old/male	HFD:7.5% saturated fat and 1.25% cholesterol	40 weeks	/	Runx2, osteocalcin, and caspase‐3 upregulated Wnt/β‐catenin pathway activation	AV area decreased Transvalvular pressure gradient and flow velocity increased LVEF reduced	/	/	[51]
		8 weeks old/male C57BL/6J background	HFD:21% fat and 1.25% cholesterol or after add 1.5% adenine at 12 weeks	12 to 24 weeks	Aortic valve thickening Col1A1 increased Macrophage increased	ICAM‐1 levels elevated	Peak aortic valve velocity and area unchanged at 12 weeks Velocity increased and area reduced at 24 weeks	Uric acid increased	/	[48]
Rabbits	New Zealand white rabbits	Male/weighing 2.5–3.0 kg	HFD containing 0.5% cholesterol with 25 000 IU of vitamin D2 daily	12 weeks	Aortic valve leaflet thickening Myocardial fibrosis increased Calcification. α‐SMA staining elevated Macrophage infiltration increased	/	/	/	Transaortic maximal velocity and mean transaortic pressure gradients increased	[26]

Additionally, while diet‐induced animal models easily and directly reflect early CAVD via alterations in nutritional profiles, they are limited by relatively long induction periods, often exceeding 6 months. More significantly, metabolic differences exist between species, and mouse and rabbit models usually lack the kind of focal calcific lesions seen in humans.

#### 2.1.2 Genetically modified animal models of CAVD

Genetically engineered animal models are constructed through gene mutation, knockout, or transgenic techniques to achieve the acquisition or deletion of specific gene functions. Mice are the most commonly used animals in CAVD research due to the availability of mature gene editing methods and efficient breeding systems. Rather than list all types of models individually, Table 3 highlights representative mouse models to elucidate the core pathogenic mechanisms of this disease.

**TABLE 3:** Genetically modified mouse models of aortic valve calcification.

Modified gene	Strain/gene	Age/gender/weight	Method		Phenotypes					References
			Model construction	Feeding time	Pathological phenotype	Molecular markers	Ultrasonic cardiogram	Metabolic parameters	Hemodynamic parameters	
ApoE^−/−^	C57BL/6J background	4 weeks old	AAV2‐shRNA tail vein injection followed by 0.25% high cholesterol diet	24 weeks	Aortic valve leaflet thickening Collagen accumulation Calcium deposition	/	Peak transvalvular jet velocity increased Mean transvalvular pressure gradient increased AVA decreased	/	/	[62]
ApoE^−/−^ *Gch*1^−/−^	/	8 weeks old	HFD after gene knockout	24 weeks	Severe calcification Atherosclerosis Collagen deposition	BH4 and BH2 decreased in aorta and plasma Runx2, Osterix, Notch‐1, and TLR4 upregulated VCAM‐1 and ICAM‐1 increased in CD31^+^ endothelial cells α‐SMA elevated in aortic valve leaflets. ROS and 3‐NT increased	Peak transvalvular jet velocity increased			[57]
*L* *dlr* ^−/−^	C57BL/6 background	6–8 weeks old/male	HFD after gene knockout	24 weeks	Aortic valve leaflet thickness Fibrotic area increased Calcification	RUNX2 and BMP2 upregulated Macrophage infiltration (CD68^+^) Aortic valve mitochondria reduced (TOMM20)	Peak aortic jet velocity and mean transvalvular pressure gradient increased	/	/	[58]
*Notch*1^+/−^	C57BL/6 background	/male	Western diet after weaning	10 months	Calcification	Bmp2 in the valve leaflets increased	/	/	/	[59]
ChmI^−/−^	C57BL/6 background	90.2 ± 7.4 week‐old	Gene targeting ChmI	/	Calcification Inflammation elevated (Mac‐1) Lipid deposition Aortic valve thickening Neovascularization enhanced Capillary increased	Vegf‐A upregulated	AVA increased from 32 weeks	/	/	[60]
CCN3	Myeloid‐specific CCN3 knockout mice	8–10 week‐old	Mye‐CCN3‐ko mice with LDLR knockdown were fed a HFD to induce hyperlipidemia	40 weeks	Calcification Aortic valve thickening Collagen deposition α‐SMA increased	CD68, Runx2 and BMP2 increased	Peak velocity, gradient and VTI elevated Ejection fraction decreased Left ventricular mass increased in male mice	Serum lipids unchanged	Serum unchanged	[61]

In the early 1990s, Zhang et al.^52^ and Plump et al.^53^ pioneered ApoE^−/−^ mouse models, which have become foundational models for hypercholesterolemia‐induced atherosclerosis. Shortly thereafter, Ishibashi et al.^54^ established the LDL receptor‐deficient mouse (*Ldlr* ^−/−^) strain. Typically developed from C57BL/6 mice, both models are essential for atherosclerosis and genetic interventions research. *ApoE* ^−/−^ mice are more widely used because they spontaneously develop hypercholesterolemia and rapidly form aggressive plaques without a specific diet, making them suitable for studying VLDL/LDL levels and the remnant lipoprotein metabolism. In contrast, *Ldlr* ^−/−^ mice require dietary induction to develop slower pathological changes, making them more suited for studying LDL metabolism and diet‐induced atherosclerosis.^55^ However, the plasma lipoprotein profiles of *ApoE* ^−/−^ mice differ significantly from those of typical human atherosclerosis, which is primarily driven by LDLs. While patients with ApoE dysfunction typically present cholesterol levels between 300 and 600 mg/dL,^53^ HFD‐fed ApoE^−/−^ mice exhibit severe hypercholesterolemia (cholesterol levels >1800 mg/dL) largely because of species‐specific metabolic differences.^53^ Alternatively, *Ldlr* ^−/−^ mice accumulate cholesterol mainly in the form of LDLs, more accurately reflecting human familial hypercholesterolemia.^56^

Endothelial‐specific *Gch1* knockout on an ApoE^−/−^ background (ApoE^−/−^ *Gch1* ^−/−^) allowed identification of the link between tetrahydrobiopterin (BH4) deficiency and valvular calcification: pathologically, disease progression in this model begins with endothelial activation and oxidative stress; under this microenvironment, VIC osteogenesis, ECM remodeling, calcification, and ultimately valve dysfunction develop.^57^ Mechanistically, endothelial *Gch*1 deficiency impairs BH4 synthesis, triggering eNOS uncoupling and peroxynitrite accumulation, the latter mediates DRP1 nitration, which directly drives the observed VIC osteogenesis and valvular calcification.^57^ HFD‐fed *Ldlr* ^−/−^ mice develop characteristic CAVD phenotypes accompanied by upregulated protein tyrosine phosphatase 1B (PTP1B) expression in the aortic valve. In this model, PTP1B mediates hyperlipidemia‐driven calcification by disrupting mitochondrial homeostasis, as calcification is associated with mitochondrial depletion, and PTP1B inhibition preserves mitochondrial dynamics.^58^ Although these mice successfully reproduce early human CAVD, they rarely progress to hemodynamically significant stenosis. To overcome this limitation, double‐mutant models (*Ldlr* ^−/−^/ApoB^100/100^), detailed later in this review, have been developed.

Activation of the Notch1/Bmp2/Runx2 signaling pathway exacerbates aortic valve calcification.^59^ These mice do not exhibit the typical aortic stenosis characteristic of human CAVD. However, this molecular pathway remains involved in the valve calcification process.^59^ Chondromodulin‐I (ChmI) is an elastin‐binding glycoprotein critical for elastic fiber assembly and negative regulation of TGF‐β signaling. Functional abnormalities of this protein gene can lead to ECM dysregulation, resulting in aortic valve pathology. Notably, early lesions are observed only in aged animals.^60^ The myeloid CCN3‐deficient mouse model exhibits a gender‐specific difference: male mice show significantly increased left ventricular mass, whereas females show no change, with no metabolic alterations.^61^ Mechanistically, myeloid CCN3 deficiency enhances BMP2 secretion from macrophages, and then BMP2 activates Runx2 signaling in VICs via paracrine action, driving osteogenic differentiation and calcification.^61^

Genetically engineered models provide precise tractability and reproducibility for mechanistic studies, but their prolonged timelines, technical complexity, and species differences limit their translational fidelity. While models with combined knockout of multiple genes can accelerate aortic valve stenosis modeling, they induce severe hypercholesterolemia and premature mortality, confounding longitudinal studies and failing to reflect the mild hyperlipidemia typical of early human valvulopathy. Therefore, despite their value for target validation, these models require careful interpretation of the constructed cardiometabolic phenotypes.

#### 2.1.3 Surgery‐induced CAVD models

Calcification models can be established in 28 days via subcutaneous xenotransplantation of porcine aortic valve leaflets into 3‐week‐old male Sprague–Dawley rats. Pathologically, this model recapitulates human end‐stage calcific degeneration through three key manifestations: microcalcifications, CD68^+^/CD163^+^ macrophage infiltration with upregulated TNF‐α/IL‐1β expression, and caspase‐3‐mediated apoptosis‐triggered RUNX2^+^ osteogenic transformation.^63^, ^64^ While the establishment of this inflammatory–osteogenic cascade better mimics ECM pathologies than genetic models, the resulting model is critically limited by the absence of hemodynamic shear stress, requiring combinatorial approaches with hyperlipidemic diets or mechanical loading to address translational gaps.

#### 2.1.4 Combined CAVD models

Combining genetic modification with dietary induction remains the primary strategy for modeling CAVD (Table 2). By impairing cholesterol efflux in ApoE^−/−^ mice, atherogenic diets rapidly accelerate the pathogenesis of calcification processes. For example, feeding ApoE^−/−^ mice an HFD consisting of 1.25% cholesterol, 7.5% saturated fat for 10 months triggers hemodynamically significant CAVD.^51^ Similarly, ApoE^−/−^ mice fed an HFD consisting of 42% fat and 0.2% cholesterol for 6 months demonstrate exacerbated metabolic dysfunction, increased aortic transvalvular velocity and severe calcification.^47^ Although these models are undeniably valuable for therapeutic screening, they exhibit notable limitations, including nonphysiological LDL elevation, the absence of fibrotic preconditioning prior to calcification, and an inability to replicate age‐dependent clinical progression. In weaned C57BL/6 males, 10 months of a Western diet containing 21% fat, 0.15% cholesterol drives pathogenesis, primarily through *Notch*1^+/−^‐induced repression of RUNX2 osteogenic signaling. Histologically, mutants develop valvular calcification but lack hemodynamically significant stenosis; alternatively, they may develop bicuspid aortic valves (BAVs) that are phenotypically attenuated relative to those of humans with CAVD. The limitation of this model is its inability to replicate the hemodynamic disturbances observed in human patients, as well as the compensatory left ventricular hypertrophy typically associated with chronic valvular obstruction.^59^

A mouse model combining *Ldlr* deficiency (*Ldlr* ^−/−^), a high‐fat/high‐cholesterol (HF/HC) diet and an aortic valve wire injury intervention mechanistically integrates atherogenic dyslipidemia, hemodynamic insult, and genetic susceptibility, which synergistically drive an accelerated calcific pathogenesis process in which the LDL receptor deficiency impairs cholesterol clearance, the HF/HC diet, containing 1.25% cholesterol, 0.5% cholic acid, amplifies oxLDL deposition, and endothelial denudation from the WI initiates inflammatory–fibrotic cascades.^65^ While the model offers unprecedented recapitulation of the human‐like multihit progression of CAVD, it is limited by inconsistent WI depths and an attenuated left ventricular hypertrophy phenotype versus that observed in clinical end‐stage disease.

DUSP26 is highly expressed in human calcified aortic valves, where it mediates MAPK/NF‐κB hyperactivation.^64^ This process can be modeled in 4‐week‐old male ApoE^−/−^C57BL/6 mice administered AAV2‐sh‐DUSP26 via tail vein injections and maintained on a 0.25% HCD for 24 weeks.^62^ Pathological progression manifests as severe, calcific AVS with left ventricular obstruction, pressure‐overload hypertrophy, and heart failure but without significant chamber dilation because of an insufficient duration of hemodynamic stress.^62^ While this model leverages the experimental tractability of mice for rapidly validating treatment targets, its translational relevance is constrained by the nonphysiological hypercholesterolemia that is induced (serum LDL >500 mg/dL *vs*. human LDL 130 ± 40 mg/dL) and the incomplete end‐stage fibrocalcific remodeling.^62^

A multimodal CAVD model can be developed by combining genetic, viral, and dietary interventions. Injection of 8‐ to10‐week‐old myeloid‐specific CCN3 knockout mice with rAAV8/D377Y‐mPCSK9 induces sustained *Ldlr* knockdown and hyperlipidemia, which can be further potentiated through administration of an HFD for 40 weeks.^61^ The model robustly exhibits the pathological hallmarks of CAVD, including progressive AVS with concomitant systolic impairment and minimal aortic regurgitation (AR).^61^ While this combinatorial strategy offers excellent pathophysiological recapitulation of human CAVD through the combination of genetic susceptibility, acquired dyslipidemia and chronic metabolic stress, its utility is limited by its relatively long developmental timeline, technical complexity, and high resource requirements.^61^

### 2.2 Animal models of aortic valve stenosis

Aortic valve stenosis (AVS) is characterized by progressive left ventricular outflow obstruction that stems from hemodynamic‐induced endothelial dysfunction, maladaptive VIC activation, and dysregulated ECM remodeling.^24^, ^66^ The pathological trajectory initially involves fibro‐calcific leaflet thickening driven by the osteogenic differentiation of VICs. This process is potentiated by proinflammatory cytokines, dysregulated calcium‐phosphate metabolism, and aberrant mechanotransduction, culminating in reduced mobility, elevated transvalvular pressure gradients, and compensatory left ventricular hypertrophy. While both AVS and AR result from hemodynamic alterations, AR typically follows an intrinsic aortic root pathology or systemic inflammatory conditions; thus, this review focuses on AVS models. Contemporary modeling has evolved from surgical constriction to genetically engineered platforms that mimic human etiologies, diet‐induced metabolic injury, and combination systems. These models allow the mechanistic identification of stage‐specific events and provide platforms for evaluating therapies targeting calcification or ECM preservation.^67^

#### 2.2.1 Diet‐induced AVS models

Diet‐induced AVS models primarily reflect the hypercholesterolemia‐driven pathophysiological cascade, wherein dysregulated lipid metabolism initiates valvular endothelial dysfunction, promoting lipoprotein retention and oxidative stress that subsequently induce pro‐osteogenic differentiation in VICs. Table 4 lists recent HCD‐ and HFD‐based models of AVS.

**TABLE 4:** High‐cholesterol and high‐fat model of aortic valve stenosis.

Diet	Species	Strain/gene	Age/gender/weight	Method		Phenotypes					References
				Dietary components	Feeding time	Pathological phenotype	Molecular markers	Ultrasonic cardiogram	Metabolic parameters	Hamodynamic parameters	
HCD	Mice	C57BL6/J mice	4 months old	1% cholesterol Western diet	8–12 months	Valve thickening. No evident surface calcification	αSMA increased	Peak velocity and mean pressure gradient elevated	/	/	[68]
	Rabbits	New Zealand white rabbits	Male/2.5–3.0 kg	1% cholesterol diet	8 weeks	Lipid infiltration Collagen deposition Macrophage infiltration (RAM 11) Myofibroblast PCNA staining increased Osteopontin expression elevated	PCNA cells increased Osteopontin and Cbfa‐1 upregulated	/	/	/	[70]
		New Zealand white rabbits	12–13 weeks old/male/2.7–3.0 kg	0.5% cholesterol diet with vitamin D_2_ (50 000 IU/day) in drinking water	13 weeks	Calcification Reduced AVA Valve thickening	/	/	/	/	[74]
	Pigs	Yorkshire Barrow pigs	3 months old	A diet containing 12% lard, 1.5% cholesterol and 32% total fat	2 weeks‐6 months	2 weeks: Lipid deposition and calcific nodules on aortic side 6 months: Severe lipid deposition with focal aortic‐side calcification No significant inflammation	PPARγ increased in endothelial cells	/	/	/	[72]
HFD	Mice	C57BL/6N mice	6‐week‐old/male	HFD:31.5% lard, 3.1% soybean oil, 8.5% saccharose and 15.6% maltodextrin	7 months	Calcification. Left ventricular wall, septal, and aortic valve thickness increased	AGE and RAGE upregulated	Systolic transvalvular velocity increased Left ventricular and AVA decreased	/	Serum total cholesterol, triglycerides, and LDL elevated	[73]

In C57BL/6J mice, a Western diet containing 1% cholesterol resulted in the recapitulation of classic features of AVS.^68^ However, while this model exhibits certain phenotypic similarities to AVS, it fails to accurately reflect the stages of disease progression observed in elderly human AVS patients.^68^

New Zealand white rabbits consistently fed an HCD develop early‐stage AVS.^69^ Analysis of this model demonstrates that hypercholesterolemia drives concurrent atheromatous proliferation and osteogenic differentiation.^70^ However, the model's utility is limited by severe, survival‐compromising hepatotoxicity and relatively long induction timelines. Crucially, an HCD alone induces only valve stiffening; vitamin D must be coadministered to drive definitive stenosis and severe calcification.^71^

In porcine models, inducing hypercholesterolemia through a dyslipidemic diet can recapitulate the early pathogenesis of aortic valve sclerosis. By administering dietary interventions for durations ranging from 2 weeks to 6 months, this model achieves pathologically relevant elevations in total and LDL cholesterol levels (>300 mg/dL and >200 mg/dL, respectively), leading to proteoglycan‐rich fibrotic plaques within the valve fibrosa.^72^ Although this model effectively recapitulates side‐specific, early pathological progression consistent with that of human AVS, key translational limitations remain: the model fails to progress from sclerosis to clinically significant calcification. Additional limitations include poor characterization of deeper VIC activation mechanisms, slow disease progression, and high resource demands, all of which limit experimental throughput.

C57BL/6N mice fed a 7‐month HFD recapitulate typical valvular stenosis, accompanied by systemic metabolic disturbances.^73^ At the cardiac level, pressure‐overload adaptation is observed, manifesting as left ventricular wall and septal thickening and a reduced chamber area, which are consistent with the characteristics of ventricular hypertrophy. That study provides the first evidence that a prolonged (7 months) HFD in wild‐type mice can yield a complete pathological sequence from metabolic dysfunction to valvular calcification/stenosis and cardiac remodeling.^73^

#### 2.2.2 Genetically modified AVS models

LDLR is a hepatic transmembrane glycoprotein that mediates the endocytic clearance of LDLs and VLDLs, critically regulating plasma cholesterol homeostasis. *Ldlr* ^−/−^ mice expressing only apolipoprotein B100 (*Ldlr* ^−/−^A*po* *b* ^100/100^, or “LA mice”) are prone to developing calcifying AVS.^18^ Pathologically, LA mice exhibit increased endogenous cholesterol levels, accompanied by the spontaneous development of calcific AVS within 20–30 weeks, elevated transvalvular pressure gradients (>40 mmHg), progressive valve leaflet thickening with microcalcifications, and histological evidence of lipoprotein retention and osteogenic differentiation.^18^ This model recapitulates human‐like calcific disease progression and the associated hemodynamics but requires extended maintenance for phenotype manifestation; concurrent atherosclerosis further complicates the analysis of isolated valves, and the husbandry costs are high.^18^

Disruption of epidermal growth factor receptor (*EGFR*), a tyrosine kinase that regulates valvulogenesis via ERK signaling pathways, via alteration of the hypomorphic *Egfr* allele models calcific AVS.^74^ Mutant mice generated on a C57BL/6J (B6) or 129S1/SvImJ (129) background exhibit striking background‐dependent expression.^74^ Models bred on the B6 background demonstrate that VIC proliferation, macrophage infiltration and collagen disorganization drive severe calcific aortic stenosis, supporting the progression from congenital leaflet thickening to valve dysfunction, compensatory hypertrophy, and eventual heart failure. Conversely, mutants bred on the 129 background exhibit attenuated phenotypes with delayed calcification, which is attributed to differential ApoE expression modulating lipid retention.^75^ While the B6‐background model uniquely recapitulates human‐like end‐stage calcific stenosis, the utility of the models is limited by high perinatal mortality, background‐specific phenotype variability, and the absence of diet‐responsive disease acceleration.^74^, ^75^

Fibulin‐4 stabilizes the valvular architecture by crosslinking fibrillin‐1 and organizing elastin fibers within the ECM.^76^ Fibulin‐4^−/−^ hypomorphic mice are generated on a C57BL/6 background via Cre–loxP recombination followed by >N10 backcrossing; the resulting models exhibit sustained ECM dysregulation. This model recapitulates early‐stage AVS pathology, manifesting as compensatory aortic leaflet thickening, ECM disorganization characterized by elastic lamina fragmentation and abnormal collagen deposition, mild hemodynamic obstruction and ascending aorta dilation, and notably significant calcification.^77^ While the model is valuable for elucidating Marfan‐like matrix‐dependent stenosis and secondary leaflet remodeling, its utility is limited by the absence of mineralization, confounding aortic aneurysms, and subtle hemodynamic changes that can only be detected via high‐resolution ultrasound.

#### 2.2.3 Surgically induced AVS models

##### Wire‐induced models

In wire‐induced AVS models, ultrasound‐guided mechanical injury is performed to trigger maladaptive valvular remodeling. This technique involves the application of graded wire traction to progressively impose hemodynamic stress, initiating a sequence of pathophysiological responses: initially endothelial denudation promotes macrophage infiltration and collagen deposition within 4 weeks; this process eventually progresses to fibrocalcific leaflet thickening and hemodynamic dysfunction by 8–12 weeks.^16^, ^78^ The pathological progression in this acute injury model is characterized by an elevated transvalvulary velocit with left ventricular dilation and reduced ejection fraction, which corresponds to the various stages of human disease ranging from compensatory hypertrophy to decompensated failure.^16^ While this model offers advantages for studying stage‐specific mechanisms, it fails to replicate the chronic mechanical stress that persists for decades underlying clinical AVS.

##### Surgical valve replacement models

The valve replacement surgical model creates controllable hemodynamic stenoses by altering animal anatomical structures, enabling transformative evaluation of transcatheter aortic valve replacement (TAVR) devices. In ovine models, external banding above the valve induces a noncalcific stenosis, providing sufficient radial fixation for TAVR devices, with observation for 6 months.^79^ This model can simulate the acute hemodynamic obstruction state required for chronic TAVR evaluation, but its artificial nature inherently cannot replicate the typical chronic calcific degenerative features of valvular leaflets in human disease.^79^

In a porcine model, PVL is induced via balloon‐assisted commissural laceration.^80^ This model enables evaluation of the hemodynamic and energetic impacts of mild–moderate aortic regurgitation on left ventricular function and local blood flow.^81^ However, this model faces significant technical constraints; unlike the human subendocardial network, the widespread nature of the porcine Purkinje system increases susceptibility to ventricular fibrillation and sudden decreases in aortic and left ventricular pressures, necessitating complex anesthetic management. Additionally, the highly variable hemodynamic compensation among individuals complicates endpoint standardization. Despite these limitations, the porcine platform remains invaluable for investigating the acute hemodynamics of mild–moderate PVL without the need for chronic calcification.^80^, ^82^

#### 2.2.4 Combined induction models

##### Genetic modification and dietary induction

In the *Ldlr* ^−/−^ *Apo* *b* ^100/100^ murine model, hypercholesterolemia drives AVD progression. In the early stages, the pathology is characterized by elevated superoxide levels, myofibroblast activation, and increased phospho‐Smad2 levels, followed by collagen deposition and colocalization with lipid plaques, as well as increased phospho‐Smad1/5/8 levels and the upregulated expression of osteogenic genes *Msx*2, *CBFA*1, and *Osterix*, ultimately resulting in significant valve mineralization and calcification.^83^ In the late stage, the pathology exhibits reduced aortic valve orifice areas but no further increase in oxidative stress or valvular lipid content. Notably, early lowering of cholesterol levels effectively slows the progression of stenosis but does not reverse the fibrosis, suggesting that late‐stage fibrosis may not be the primary cause of hemodynamic obstruction.^83^, ^84^ Analysis of the conditionally inducible Reversa model (*Ldlr* ^−/−^/*Apo* *b* ^100/100^/*Mttp*ᶠˡ^/^ᶠˡ/Mx1*Cre* ^+/+^) further demonstrates that targeted normalization of cholesterol levels via microsomal triglyceride transfer protein (Mttp) reactivation halts AVS progression,^84^ highlighting the translational potential of early lipid‐lowering interventions.^83^ While highly valuable for evaluating lipid‐targeted therapies, these models are constrained by complex breeding requirements and relatively long experimental timelines.

##### Surgical and pharmacological induction

A representative combination surgical and pharmacological model employs WI to mechanically disrupt the aortic valve leaflets in male C57BL/6 wild‐type mice immediately followed by subcutaneous high‐dose vitamin D (500 000 IU/kg/day for 3 days),^85^ leading rapidly to calcific aortic valve stenosis (CAVS). Histologically, the leaflets exhibit marked thickening with progressive collagen deposition and calcification, along with elevated levels of markers for osteogenic differentiation (Osterix), inflammation (CD68), and fibrosis (α‐SMA). Hemodynamically, the model results in significantly elevated peak aortic jet velocity and mean transvalvular pressure gradients.^85^ Key advantages of this model include rapid disease onset, low costs, genetic background independence, and suitability for large‐scale therapeutic screening. However, the results of the acute mechanical injury fundamentally differ from those due to the chronic hemodynamic stress that drives human CAVS.

##### Hyperlipidemia and mechanical injury

To accelerate AVS modeling, researchers have combined hyperlipidemia with mechanical injury. In a representative approach, surgical wire injury in ApoE^−/−^ mice leads to rapid disease progression. Pathologically, initial endothelial disruption, lipid deposition, and oxidative stress trigger inflammation (MCP‐1, Vcam‐1) and apoptosis; this inflammatory environment promotes progressive collagen deposition and subsequent fibrosis via myofibroblast activation. The fibrotic remodeling is followed by osteogenic differentiation (Runx2) and overt calcification. This cascade leads to marked leaflet thickening, which hemodynamically manifests as significantly increased transvalvular flow velocity.^86^ The pathological changes in this model are confined to the leaflet border, unlike human AVS, which typically begins on the aortic surface. Moreover, no significant cardiac dysfunction is observed, thereby limiting its clinical translational potential.

### 2.3 Animal models of aortic valve malformation

BAV is the most common congenital cardiac defect, affecting 0.5% to 2% of the population with a 2:1 male‐to‐female predominance. Anatomical subtypes can be classified by cusp fusion: right‐left (R‐L, the most common), right‐noncoronary (R‐N), and left‐noncoronary (L‐N). Other less frequent malformations include unicuspid and quadricuspid valves.^87^

*Gata6* is a transcription factor that regulates cardiac outflow tract septation, and is essential for valve development.^88^ *Gata6* ^+/−^ mice on a C57BL/6 background exhibit R‐L BAVs. Pathological features include increased collagen deposition within the ECM, valve leaflet thickening and progressive stiffening.^88^ This model also develops male predominance, mirroring human epidemiological patterns. *Gata5* ^−/−^ mice also develop similar BAVs with mild stenosis and compensatory left ventricular hypertrophy.^89^ These models are limited by the lack of chronic hemodynamic stress progression that is typical of human disease.

Discoidin, CUB and LCCL domain containing 2 (DCBLD2), an adaptor protein that regulates endocytosis and TGF‐β/BMP signaling, is critical for valvular morphogenesis.^90^ *Dcbld2* ^−/−^ mice spontaneously show BAVs with high penetrance and calcification patterns closely recapitulating those in human pathology.^90^ This model also exhibits age‐progressive microcalcification within the aortic valve, significant hemodynamic stenosis, and spontaneous lobular calcification, which is likely mediated by the aberrant cusp number and greater BMP2 signaling due to *Dcbld2* loss.^90^ While the model provides a robust platform for studying BAV‐associated calcification dynamics, it exhibits translational limitations such as the significantly lower maximum standardized uptake values among murine aortic valves than among those of humans, which is attributable to the smaller valve dimensions and differential cardiac motion artifacts during imaging.

ADAMTS16, a metalloproteinase critical for ECM organization during valvulogenesis, is also involved in BAV pathogenesis. Mouse models deficient in Adamts16 bred on a C57BL/6J background exhibit R‐N BAVs with noncoronary leaflet hypoplasia evident at E16.5; postnatally, these mice develop localized aortic valve thickening, progressive ECM disorganization, age‐related calcification, and, eventually, stenosis.^91^ While valuable for studying ECM‐mediated malformation mechanisms, these models have translational limitations, including low BAV penetrance (≤28%) and attenuated hemodynamic stress responses.^91^

Mutations in the human *NOTCH1* gene cause the development of BAVs as well as severe valvular calcification. The VEC‐targeted *Notch1* knockout model effectively recapitulates this disease progression^92^: embryonic deletion of *Notch1* induces outflow cushion overfusion, aortic sinus hypoplasia, and abnormal coronary origins, resulting in BAV development.^92^ Mechanistically, the Notch1/TNF signaling axis balances interstitial cell proliferation and apoptosis after EMT; *Notch1* deficiency disrupts this homeostasis to drive both maldevelopment and adult calcification.^92^ Although the model is valuable for elucidating immune and metabolic mechanisms, it is limited by fundamental differences between murine and human hemodynamic stress environments. Heterozygous *Egfr* ^Vel/+^ mice simulate human unicuspid aortic valve pathology, and 79% of juveniles exhibit this condition. Advanced severe valvular malformations can lead to moderate to severe aortic regurgitation and stenosis. These hemodynamic changes can lead to compensatory left ventricular dilation and cardiac hypertrophy.^80^ This progressive course reflects the clinical manifestations in young patients, ultimately progressing to valvular cardiomyopathy.^93^ The model is limited by differences in murine hemodynamic adaptation and an inability to accurately reflect human afterload responses.

### 2.4 Animal models of hereditary aortic valve dysplasia

Hereditary aortic valve dysplasia can be studied using animal models with natural genetic mutations, providing critical insights into its pathogenesis. These animal models spontaneously develop calcification that simulates human pathological features, offering a physiologically relevant platform for studying disease progression. In contrast, murine models require long‐term experimental induction to achieve comparable calcification phenotypes.^94^

Watanabe heritable hyperlipidemic (WHHL) rabbits carry a natural *Ldlr* mutation that causes lifelong hypercholesterolemia without the need for dietary intervention, allowing the modeling of congenital dyslipidemia. Valve thickening, matrix accumulation, and osteogenic transition can be accelerated in juvenile males with a 24‐week diet of 0.25% cholesterol.^32^ Conversely, aging WHHL rabbits fed a normal diet spontaneously develop AVS by 30 months.^93^ Compared with wild‐type rabbits fed dietary cholesterol for short durations, WHHL rabbits maintain chronic, mild hypercholesterolemia, which better reflects human disease progression.^95^ Furthermore, their lipoprotein profiles are more similar to those of humans, as liver toxicity caused by dietary cholesterol is avoided. However, despite the significant AVS, this model lacks left ventricular hypertrophy, indicating altered secondary remodeling.

Rapacz familial hypercholesterolemic (RFH) swine exhibit persistent hypercholesterolemia even on standard diets.^96^ This model demonstrates stage‐distinct valve pathology: at 1 year, aortic leaflets exhibit ECM remodeling with lipid oxidation; by 2 years, macrophage infiltration and complex atherosclerotic lesions have occurred; and most interestingly, at 3 years, despite mild leaflet thickening, the aortic valves maintain near‐normal hemodynamics.^96^ RFH pigs can be useful for studying the onset and early progression of CAVD without the need to induce advanced AVS or calcified valvular disease.^96^ Key advantages of this model include its physiological similarity in terms of human cardiovascular dimensions, the absence of confounding comorbidities, and predictable age‐dependent pathology. However, the exclusive use of female cohorts due to management constraints cannot capture the twofold higher CAVD risk in human males, potentially limiting translational generalizability.^96^

## 3 APPLICATION OF ANIMAL MODELS IN AVD RESEARCH

### 3.1 Elucidating pathogenetic mechanisms and therapeutic targets

The absence of disease‐modifying pharmaceuticals for treating aortic valve pathologies underscores the critical need for mechanistic insights from animal models. HFD‐fed ApoE^−/−^ murine models of aortic valve calcification have served as indispensable platforms for mechanistic discovery and therapeutic validation, the investigations of which have yielded three pivotal therapeutic breakthroughs. First, andrographolide has been shown to suppress or inhibit VIC differentiation through the Ccnd1/Trim25/Nrf2 signaling pathway,^97^ thereby attenuating calcification progression. Second, in AVCAPIR^−/−^ApoE^−/−^ double knockout mice, disruption of CD36 demethylation activated the pro‐calcific CD36/PCSK9 cascade, revealing a dual diagnostic/therapeutic target.^98^ Third, morusin, a prenylated flavone, alleviates aortic valve calcification in HFD‐fed ApoE^−/−^ mice, a result attributed to its antioxidant and anti‐senescence effects that ultimately inhibit osteogenic VIC differentiation through the Ccnd1/Trim25/Nrf2 pathway.^97^ In HFD‐fed ApoE^−/−^ murine models, the therapeutic effects of methods ranging from metabolic reprogramming to RNA epigenetics and oxidative stress pathway regulation have been further explored. Thus, the use of genetic mouse models has not only helped decipher the fundamental mechanisms underlying CAVD but also accelerated the clinical translation of three promising disease‐modifying therapeutic strategies.

The construction of complementary dyslipidemic models has further expanded the CAVD therapeutic landscape. In *Ldlr* ^−/−^ mice subjected to wire injury, PTP1B inhibition attenuates aortic valve calcification and stenosis by restoring OPA1‐mediated mitochondrial homeostasis during VIC osteogenesis, revealing a drug‐treatable metabolic target.^58^ In C57BL/6 mice fed a diet supplemented with choline (a TMAO precursor), the addition of an HFCD elevates circulating TMAO levels, which contributes to aortic valve calcification, suggesting that dietary intervention may prevent TMAO‐induced calcification.^42^ In HFD‐fed myeloid‐CCN3‐ko mice, myeloid CCN3 alleviates aortic valve calcification by regulating macrophage BMP2 secretion.^61^ In a hamster CAVD model, AGE‐LDL induces inflammatory and osteogenic responses in human aortic valve interstitial cells through the AGE‐LDL/RAGE/NF‐κB signaling pathway.^81^ IL‐37 inhibits this process and alleviates lesions, suggesting the targeting of this pathway as a potential therapeutic strategy.^81^

ApoE^−/−^ mice fed an HCD have been instrumental in identifying therapeutic targets for CAVD. In mice treated with DUSP26 shRNA adeno‐associated viruses and fed an HCD, DUSP26 facilitates aortic valve calcification by inhibiting DPP4 degradation, suggesting that silencing or inhibiting DUSP26 could serve as a promising therapeutic approach for halting CAVD progression.^62^ Additionally, Nesfatin‐1 effectively inhibits osteogenic AVIC differentiation by preventing ferroptosis, primarily through the GSH/GPX4 and ZIP8/SOD2 pathways.^99^ In ApoE^−/−^ mice fed an HF/HCD, Nesfatin‐1 can reduce aortic valve calcification by inhibiting osteogenic AVIC differentiation, which is likely mediated by the GSH/GPX4 and ZIP8/SOD2 pathways.^99^

Genetically modified mice serve as crucial animal models for studying CAVD. DPP‐4 inhibitors (e.g., evogliptin) inhibit calcified nodule formation and alleviate AVS symptoms in eNOS^−/−^ mice and HCD‐fed rabbits, potentially delaying calcification.^62^ When small nucleolar RNA host gene 3 (SNHG3) is silenced in ApoE^−/−^ mice, aortic valve calcification is attenuated, and the BMP2 pathway is enhanced, suggesting that SNHG3 is a novel target of pro‐osteogenic differentiation in CAVD.^100^ ATX inhibition has been shown to decelerate fibrocalcific aortic valve disease (FCAVD) progression and prevent fibrosis and calcification in VICs, thereby suggesting the potential of ATX inhibitors in the treatment of FCAVD.^40^

Despite these advances, the targeted delivery of therapeutics for CAVD remains a significant challenge. In an HFD‐fed *Ldlr* ^−/−^ mouse model, nanocarriers were used to deliver the anti‐calcification agent XCT790 to calcified aortic valves. This process effectively inhibited osteogenic differentiation among valvular stromal cells and alleviated both aortic valve calcification and stenosis.^101^ These findings underscore the critical role of animal models in the development and evaluation of efficient drug delivery systems, as they serve as vital tools for drug screening and the exploration of potential therapeutic interventions.

### 3.2 Use of animal models for facilitating the clinical application of TAVR devices

To date, no effective targets for preventing and treating AVD have been identified, and surgical intervention remains the primary management approach. Over the past decades, however, TAVR has proven to be an effective treatment modality for patients with high‐risk AVS. Still, it is crucial to assess the feasibility, efficacy and safety of TAVR devices through the use of animal models and to determine the optimal surgical position. Notably, animal models employing sheep or pig hearts can replicate human anatomical structures well.^80^

Ovine models, which exhibit comparable aortic annular dimensions (19–22 mm) and hemodynamic profiles to those of humans, allow the rigorous validation of transcatheter technologies. The novel adjustable annuloplasty procedure establishes reproducible AVS in sheep, facilitating longitudinal evaluation of TAVR device durability and hemodynamic performance for more than 24 months.^79^ Buszman et al.^102^ surgically induced stenosis via circumferential ligation in a sheep model, and then implanted a transcatheter balloon‐dilatable valve at the left carotid annular band and conducted a 6‐month follow‐up to assess the outcomes of the procedure. This ring‐tied sheep model provides adequate anchoring for balloon‐dilatable prostheses. However, a significant limitation is that the induced stenosis leads to insufficient prosthesis expansion, resulting in suboptimal hemodynamic performance and durability challenges.^103^ A balloon dilatable TAVI system implanted in the descending aorta of young sheep for 5 months resulted in no structural degeneration and satisfactory healing and may better address the specific requirements of TAVI procedures in low‐ and middle‐income countries than conventional systems can.^102^ A surgical ascending aortic ligation ovine model was employed to assess a novel balloon‐expandable polymer transcatheter valve. TAVI was performed 2 weeks after minimally invasive left thoracotomy ligation; at 6‐month follow‐up, the valves showed sustained function, good biocompatibility, and low mortality.^104^ In a separate model, aortic stenosis was created using annular stenosis combined with a bioengineered scaffold. By 2–4 weeks post‐surgery, marked hemodynamic changes had developed: forward peak aortic flow velocity rose, and mean transvalvular gradient increased, effectively replicating the conditions of TAVI deployment. Valves implanted in this model remained fully functional at 6 months, free from severe stenosis, clinically significant regurgitation, or major paravalvular leak.^105^ Owing to their biological characteristics, sheep provide a dependable platform for long‐term translational device evaluation and robust preclinical validation.

Porcine models have been used to assess the early feasibility of pioneering, single‐session transcatheter triple‐valve replacement using bioprosthetic devices. The procedure, which achieved an 80% acute success rate with no significant PVL, has demonstrated robust initial sealing integrity and hemodynamic stability.^106^ A preclinical study involving porcine models evaluated the novel transcatheter leaflet‐splitting device to restore coronary flow in TAVR‐related coronary obstruction; following functional and safety evaluation in pigs, the device was used in eight patients with failed bioprosthetic valves, all of whom achieved successful TAVR without obstruction.^107^ Porcine models have also been used to evaluate different delivery routes. In a study testing early feasibility of a single‐session endovascular prosthesis for the ascending aorta, aortic valve, and mitral valve, stents were implanted via transapical and transseptal approaches. The deployment was successful in 90% of the tests, with no regurgitation, coronary obstruction, or outflow compromise, confirming the feasibility of the prosthesis.^106^ These results underscore the utility of the porcine model for testing transcatheter valve technologies and provide a reliable preclinical platform for translating new devices into clinical practice.

## 4 DISCUSSION AND FUTURE PERSPECTIVES

This review synthesizes information on advances in animal models for four clinically distinct aortic valvulopathies: calcific degeneration, stenosis, malformation (particularly a bicuspid anatomy), and hereditary dysplasia. Previous researchers have developed models of AVD through approaches such as dietary interventions and genetic modification, which capture key stages of human disease progression, from early molecular alterations to end‐stage hemodynamic disturbances.

### 4.1 Anatomical and physiological characteristics of animal models

Mice are the most widely used models for AVD, primarily due to their low cost and relatively straightforward genetic modification. Under high magnification, the apical structures of human and mouse aortic valves exhibit similar ECM characteristics.^6^ Significant differences remain between the valve structures of mice and humans: (1) The valves of mice lack the characteristic three‐layer structure typical of human valve leaflets; (2) The size of the mouse aortic valve is smaller than that of the human aortic valve, which may lead to discrepancies in cardiac hemodynamic assessments; (3) The SUVmax of the mouse aortic valve is lower than that of the human aortic valve, potentially attributable to the differences in size and movement. The aortic valve of rabbits exhibits a three‐layer structure that is highly similar to that of the human aortic valve. However, there are significant differences between rabbits and humans in lipid metabolism. The porcine models have genomic homology with humans, which supports their use in aortic valve genome research.^82^ Their vascular anatomical structure, lipid profile, and lipoprotein metabolism are similar to those in humans; under a HFD/HCD, they develop atherosclerotic lesions analogous to those observed in humans, making them an ideal model for studying atherosclerosis.^108^ Conventional pigs are large and costly; miniature pigs, smaller and cheaper, provide a viable alternative and are already employed in diet‐induced atherosclerosis studies. Sheep exhibit physiological parameters that closely resemble those of humans, including heart rate, blood pressure, and aortic diameter.^79^ Sheep still present several limitations: they require longer feeding periods and have longer growth cycles.

### 4.2 Translational application and selection strategies of animal models

Given the above differences, the translational value of various animal models varies significantly. Small animals are used mainly to explore pathogenesis and therapeutic targets. As discussed in 3.1, work in these models has pinpointed pathways like mitochondrial dysfunction, epigenetic reprogramming, and inflammatory osteogenic cascades; small molecules,^58^ nutritional interventions,^42^ and biologics all show disease‐modifying potential. Translation remains difficult, however, because of differences in cardiovascular anatomy and limitations of in vitro systems. Large animals, whose anatomy and physiology more closely match humans, are the workhorses for device development and preclinical testing. As discussed in section 3.2, porcine and ovine models, through rigorous evaluation, have successfully integrated engineering advances with clinical evidence to optimize TAVR, as reflected in improved bioprosthetic valve durability,^79^ reduced PVL,^106^ and extended device longevity. This path from molecular insight to device validation has moved technologies into clinical trials and narrowed gaps in valve disease care. In the specific field of bioprosthetic valve evaluation, the ovine model is particularly important due to its unique tendency for accelerated calcification. Flameng et al.^109^ demonstrated that juvenile sheep replicate the long‐term calcification process seen clinically within 3–6 months after implantation, and the model distinguishes among multiple independent factors such as age, implant position, valve design, tissue type, and anti‐calcification treatment. On this basis, a standardized testing framework was proposed: right‐sided valved conduits should be evaluated in juvenile sheep at the pulmonary position, while stented bioprostheses are best tested in adolescent sheep at the mitral position.^109^ Building on this work, Meuris et al.^110^ introduced a simplified jugular vein tissue implantation model in juvenile sheep, finding that blood contact markedly increased calcification, whereas arterial pressure added no further effect. Together, these studies underscore the translational value of the sheep model for screening anti‐calcification strategies and probing host‐implant interactions, cementing its status as a reliable and widely used platform for evaluating bioprosthetic valve calcification and hemodynamics. Recently, Flameng et al.^111^ used ovine models to test an absorbable transcatheter heart valve made from electrospun supramolecular polymers. Designed to overcome structural degeneration and calcification of conventional bioprosthetic valves through endogenous tissue repair, the valve showed feasibility and favorable tissue integration at 12 months. This underscores the value of ovine models for evaluating next‐generation anti‐calcification materials. Consequently, model selection depends on the research objective: mouse models for molecular discovery, porcine models for translational therapy, and ovine models for device testing and anti‐calcification assessment in bioprosthetic valves.

### 4.3 Animal models drive future integration with emerging technologies

Multi‐omics technologies are increasingly integrated with animal models, reshaping heart valve disease research. Single‐cell omics in hyperlipidemic mice revealed LDL‐driven macrophage infiltration and protective PPARγ activation, offering an early intervention strategy for AVD.^23^ Multi‐omics of ApoE/LUM double‐knockout mice confirmed that LUM drives VIC osteogenesis via inflammatory pathways.^112^ Zebrafish screening combined with CRISPR/Cas9 mouse models and iPSC transcriptomics has revealed the molecular mechanisms by which ADAMTS16 deficiency drives aortic valve maldevelopment and disease progression.^91^ PET/CT and single‐cell network analysis in miR‐26b knockout mice showed that fibroblast‐derived Bmp4 paracrine activation of smooth muscle Smad1 phosphorylation induces reversible aortic microcalcification.^113^ Mouse models combined with ^18^F‐NaF PET/CT molecular imaging have further confirmed that aortic valve calcification worsens with aging and BAV severity, offering a new noninvasive tool for monitoring and intervention assessment in CAVD.^90^ Moreover, multimodal artificial intelligence models have been applied to predict arrhythmic death risk in cardiomyopathy.^114^ Looking forward, integration of single‐cell omics with CRISPR high‐throughput platforms and AI‐driven phenotyping will refine molecular classification and disease‐gene mapping. Notably, although these new technologies boost advancing the paradigm of precision medicine, they may have limitations such as an inability to model systemic neuroendocrine immune interactions or chronic hemodynamic loads, and thus cannot replace whole animal experimental systems. Combining new technologies with in vivo validation in genetically humanized animal models will constitute the next frontier in comprehensive disease modeling.

## AUTHOR CONTRIBUTIONS

**Liya Hou:** Conceptualization; writing – review and editing; writing – original draft; funding acquisition. **Xue Liu:** Conceptualization; writing – review and editing. **Wei Zhu:** Conceptualization; writing – review and editing; writing – original draft; funding acquisition. **Wangxing Hu:** Supervision; conceptualization. **Xianbao Liu:** Conceptualization; writing – review and editing; supervision; funding acquisition.

## FUNDING INFORMATION

The research work is supported by grant from Noncommunicable Chronic Diseases‐National Science and Technology Major Project (2024ZD0527100 to XB.L), the National Natural Science Foundation of China (82370374 and 81970230 to W.Z, 82271606 to XB.L, 82300497 to X.L, 82301765 to WX.H), National Science Fund for Distinguished Young Scholars of China (82425007 to XB.L), Zhejiang Province Science and Technology Innovation Leading Talents Project (2023R5236 to XB.L).

## CONFLICT OF INTEREST STATEMENT

During the writing of this study, the authors used ChatGPT to assist with grammar checking. With the aid of these tools, the authors reviewed and revised the content as needed and assume full responsibility for its accuracy.

## ETHICAL STATEMENT

This article is a review article and does not contain any new studies involving human participants or animals performed by any of the authors. Therefore, ethical approval and an ethics approval number were not required for this work.

## Data Availability

No new data were created or analyzed in this study. Data sharing is not applicable to this article.
